# “I Had No Choice. I Had to Come Somewhere” - The Journey to Assisted Living for Low-Income Older Adults

**DOI:** 10.1093/geroni/igaf122.1720

**Published:** 2025-12-31

**Authors:** Kimberly Hadson, Neha Tungaturthy, Daniel David

**Affiliations:** New York University, New York, New York, United States; New York University, New York, New York, United States; New York University, New York, New York, United States

## Abstract

Assisted living provides support for older adults to age in a home-like environment. Traditionally associated with clients paying for services “out of pocket”, New York’s Assisted Living Program (ALP) extends access to those who are Medicaid/Medicare dually-eligible. The purpose of this study is to describe the transition experience and “arrival story” of dually-eligible older adults into New York’s ALP and identify the influence of social determinants of health (SDOH). Low-income residents (n = 72), largely from underserved racial and ethnic backgrounds residing in three New York City ALPs were interviewed. Narrative thematic analysis is uncovering themes related to social relationships, and access to housing and healthcare. Preliminary results suggest individuals arrive via three trajectories: 1. Well-resourced individuals after a financial set-back 2. Life-long low-income older adults who cannot afford other care 3. Indigent and marginally housed individuals who develop stability through ALP support Many arrive after a medical or social emergency that led to a hospitalization or skilled nursing stay. Interactions between individual health and housing needs, interpersonal relationships and networks, community support, and policy factors lead people to choose ALP placement with some ambivalence. Residents reluctantly view their ALP placement as necessary; however, desire alternative options when few are available. Understanding how and why people enter assisted living and the influence of SDOH at multiple levels will support the implementation of policies and nursing care interventions in these lower resourced environments for healthy aging in a historically marginalized population.

